# How psychological contract violation impacts turnover intentions of knowledge workers? The moderating effect of job embeddedness

**DOI:** 10.1016/j.heliyon.2023.e14409

**Published:** 2023-03-11

**Authors:** Zhenhua He, Lifeng Chen, Zahid Shafait

**Affiliations:** a School of Logistics and Transportation and Tourism, Jiangsu Vocational College of Finance and Economics, Huaian, 223003, Jiangsu, China; b School of Public Affairs, Zhejiang University, Hangzhou, 310058, Zhejiang, China; c School of Business, Hangzhou City University, Hangzhou, 310015, Zhejiang, China; d College of Teacher Education, Zhejiang Normal University, Jinhua, 321004, Zhejiang, China

**Keywords:** Knowledge workers, Psychological contract violation, Job satisfaction, Turnover intention, Job embeddedness

## Abstract

When knowledge workers encounter psychological contract violations, they usually send out biased signals. Their job satisfaction decreases and their turnover intentions increase. However, in the increasingly competitive talent market, employees may not choose to leave when they encounter psychological contract violation. Based on the theoretical research results of the existing psychological contract violation, job satisfaction, turnover intention and job embeddedness, this paper analyzes the internal connections and deep relationships among the key elements by referring to the possible causes and results of the changes in the key elements. Survey technique was utilized while knowledge workers were selected as targeted respondents from specified provinces of China. A total of 392 valid questionnaires were selected by questionnaire survey. Statistical analysis was carried out by SPSS21 and AMOS23, and regression analysis was used to repeatedly verify the relationship between various elements. The results show that psychological contract violation positively predicts the turnover intention of knowledge workers, job satisfaction mediates the positive effect of psychological contract violation on turnover intention, and job embeddedness negatively regulates the positive effect of psychological contract violation on turnover intention. This study has theoretical and practical significance in enriching the theory and methods of organizational management, inspiring knowledge workers to stimulating their work potential, reducing employees' turnover intention, reducing the impact of resignation on the enterprise, and enhancing the enterprise's human resource management of knowledge workers.

## 1 Introduction

A good employment relationship can achieve a balance between employees and the lead stakeholders of organizations [1]. proposed that the nature of employment determines the quality of personal life, the economic operation, the vitality of democracy, and the degree of respect for human rights. The main goals of the employment relationship are efficiency, fairness, and the right to speak out. For individuals, working is not only an economic transaction but also an indication of respect for human life and dignity [2].

Knowledge workers have become a valuable asset for organizations in developing a market economy while playing an important role in the growth of a knowledge economy where perceptions of science and technology are changing rapidly [3,4]. With the continuous updating of knowledge, knowledge workers have brought incalculable market value and become vital to organizations and enterprises [[4], [5], [6]]. If enterprises rely solely on business contracts, they are more likely to lose control of their employees’ psychological state and increase the risk of core talent loss and affect job satisfaction, neither of which is conducive to the long-term and secure development of the enterprise [7].

The psychological contract maintains the common expectations between the enterprise and employees and mediates the balances between the development of the enterprise and the realization of the employees' careers, the remuneration and the value to the enterprise, the challenge of the organization, and the enjoyment of the employees' work [8]. It contains the common wishes of employers and employees. When employees perceive that the organization has failed to fulfill its promises and relevant responsibilities and that unfulfilled behaviors cannot be repaired on time, psychological contract violations are increasingly likely. After a psychological contract violation, employees’ sense of identity with the organization will be reduced, employees will be impelled to re-evaluate the enterprise culture, reduce their psychological reliance on the enterprise, and even resign [9].

The theory of resource preservation points out that resources constantly interact between individuals and the social environment. When an employee's loss of resources cannot be compensated in a timely and effective manner, the employee may have a negative motivation to deal with resources, including negative work attitudes and work behaviors [10]. Psychological contract violations will cause employees to endure huge psychological pressure, upsetting the individual's resource balance and causing employees' decline in job satisfaction [11]. People's perception of work itself, the environment, and other factors is expressed through job satisfaction. Meanwhile, job satisfaction, as the most direct attitude variable associated with employees' work, can predict employee turnover intentions significantly [12]. Therefore, this study plans to use job satisfaction as the mediating variables of psychological contract violation and turnover intention, respectively, to fully understand the specific ways that psychological contract violation affects employees' turnover intention.

Bambacas et al., 2013 [13] explored how management system and environment affect individual turnover intentions under job embeddedness in Chinese context. Their empirical analysis showed that job embeddedness can significantly regulate the influence of a knowledge-based management system on turnover intention. Job embeddedness is regarded as the moderating variable between the psychological contract violation and the influence of job satisfaction on the employees’ turnover intention, to explore the turnover intention of employees after the psychological contract violation occurs under different job embeddedness situations.

Based on the relevant theories of organizational behavior, this study explains the mechanism of psychological contract violation on employees' turnover intention, constructs a relationship model of different variables, and draws research conclusions through empirical tests. The in-depth study of this issue not only enriches the related research in the field of psychological contract theory and turnover intention but also provides useful enlightenment for the practice of an organization's human resource management.

## 2 Research theories and hypotheses

### 2.1 Psychological contract violation and turnover intention

According to the theory of social exchange, once employees perceive psychological contract violations, especially when they are convinced that the organization will no longer fulfill its previous commitments, they feel disappointed and frustrated with the organization. According to Ref. [14]; social exchange is an obligation demand, whereby the purpose of the employee's extra effort is to obtain a reward from the enterprise, and its essence is the principle of reciprocity [15]. fairness theory maintains that when employees find that the organization is unfair, and when they perceive that they have not been rewarded for their efforts, they experience an imbalance, and they will eventually quit their current jobs.

Ref. [16] found that psychological contract violations negatively affect employees' perception of their organizational obligations, simultaneously resulting in negative behaviors, such as resignation, decreased job performance, and decreased organizational citizenship behavior [17]. also found that psychological contract violations can lead to a decrease in employees' job satisfaction and organizational commitment, a decrease in the performance of in- and out-of-role behaviors, and an increase in willingness to leave the organization. [18]; confirmed in an empirical study of Hong Kong employees that the history of organizational changes and contract violations is related to psychological contract violations, while psychological contract violations are related to the outcome variables, including turnover intentions, indifferent behaviors, and civic virtues. [19]; proposed a psychological contract violation model that connects psychological contract violations with fairness in organizational procedures and organizational citizenship behavior. They pointed out that the various antecedent variables of psychological contract violation lead to employees' sense of unfairness, provoking emotional reactions when the psychological contract is violated. They also added that the result of this psychological contract violation is that employees’ organizational citizenship behavior decreases, and employees seek new employment opportunities elsewhere.

Therefore, this study proposes the following hypothesis.

H1 Knowledge workers' psychological contract violation has a positive effect on turnover intention.

### 2.2 Psychological contract violation and job satisfaction

There are three forms of employees' psychological contract violation: the inconsistent understanding between the organization and the employee, the organization's refusal to honor the contract, and the organization's inability to perform the contract. When these three situations occur, employees may experience a continuous process of perceiving unfulfilled promises, the breakdown of the psychological contract, and the violation of the psychological contract [20]. Each stage of this process is affected by the individual's subjective information collection and the employees' information processing.

Ref. [21] proposed the following definition of the job satisfaction conception: an emotional experience of employees of work and work-related activities. When measuring job satisfaction, it is necessary to measure not only overall satisfaction but also several key factors that constitute job satisfaction, including salary, management level, promotion, relationship with colleagues, and the job itself [22]. Several key factors that constitute job satisfaction in an organization may become triggers for psychological contract violation. Employees' dissatisfaction with a single factor may be infinitely magnified, reducing the overall evaluation of job satisfaction [23]. When a manager transferred an employee to a new position in another department, the person concerned discovered that they were not good at the position and the real situation was inconsistent with what the manager had said, thereby constituting a psychological contract violation. This negative sentiment directly led to a decline in the person's job satisfaction, and they could no longer consider other possible advantages of the organization, such as organizational reputation, promotion prospects, salary, and benefits [24].

Due to the failure of agents at all levels of the organization to fulfill their implied promises, the employee perceived violations of the psychological contract, directly leading to a decline in job satisfaction [25]. Therefore, this study proposes the following hypothesis.

H2 Employees' psychological contract violations have a negative impact on job satisfaction.

### 2.3 Job satisfaction and turnover intention

Studies have shown that low job satisfaction leads to high withdrawal behaviors, including high resignation behavior [26], high transfer behavior [27], and high willingness to resign [28]. Job satisfaction further is defined as an employee's emotional experience of work and work-related activities [22]. When employees have a low overall evaluation of an organization, they can become depressed and experience disappointment with the organization and may choose to resign [29].

Therefore, this study proposes the following hypothesis.

H3 Job satisfaction has a negative impact on turnover intention.

### 2.4 The mediating role of job satisfaction

The failure of professionals at all levels of an organization to fulfill their organizational responsibilities for intentional or unintentional reasons, and failures arising from lack of ability, cause employees to encounter psychological contract violations [30]. Unfulfilled organizational responsibilities may only be an aspect of the work content of employees, but they can become key factors in the employees' evaluation of the organization, thereby causing a decline in employees' job satisfaction and leading to turnover intention [31]. Psychological contract violations of employees lead to a decline in job satisfaction, and a loss of confidence in an organization and in the employees’ employment situation [32]. Employees may proceed to leave the organization by looking for a new job or considering resigning [33]. Therefore, this study proposes the following hypothesis.

H4 Job satisfaction plays a mediating role between psychological contract violation and turnover intention.

### 2.5 The regulative effect of job embeddedness

When employees encounter a psychological contract violation, it does not necessarily lead to their resignation. Starting from the general reality of the social embeddedness of work and life, employees will also consider practical problems related to their work [34]. Job embeddedness is like a net, allowing people to penetrate into it. People with high embeddedness have many close and distancing social connections, and they are embedded or trapped in the social network of work and life through the combination of relationships [35,36]. [37]; expressed the following view while explaining the embedded human social life pattern: the non-economic motives of human existence, such as sociality, desirability, status, and power, are also its central motives, and the realization of these motives cannot be separated from the social network.

Employees who have job embeddedness and who encounter psychological contract violations in the path of “psychological contract violation-job satisfaction-turnover intention” experience reduced job satisfaction, potentially triggering resignation [38]. However, the dimensions of job embeddedness, community, and organizational links show that there are formal and informal dependencies between individuals, institutions, and others [39]. The concept of embeddedness implies that all kinds of social relationships, including employees' work and non-work friends, groups, associations, and the natural environment in which employees live, connect employees with their families and economic networks socially and psychologically [40]. The greater the scale of the individual's connection with the network, the more constrained the dependence of employees on work and the organization. In reality, some employees would rather tolerate psychological contract violations than choose to resign [41]. One important reason is that employees seem to be bound to their organization. It is also difficult for them to leave the existing organization since they have become accustomed to the enterprise culture [42]. Some employees leave their original organizations for a while and then return to the same organization. In short, they have a strong connection with the habits of their working life and with their colleagues [43].

Therefore, this study proposes the following hypothesis.

H5 Job embeddedness plays a moderating role in the influential process of psychological contract violation on knowledge workers' turnover intention.

**Fig. 1:**
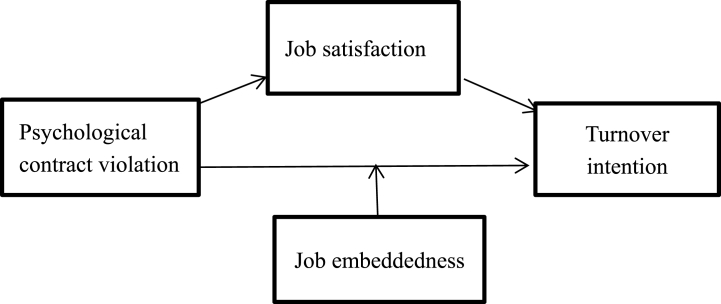
Conceptual Framework includes Psychological contract violation as independent variable; Turnover intentions as dependent variable; Job satisfaction as mediating variable and Job embeddedness as moderating variable.

Based on the above analysis, the relationship between independent variables, dependent variables, intermediate variables, and adjustment variables is shown in Fig. 1.

## 3 Research methods

### 3.1 Research samples and investigation process

To ensure the scientific validity and rigor of the survey data, the groups surveyed are all knowledge workers who are generally recognized by society and meet certain expectations regarding the content of their work and their educational background. This study used an online questionnaire to collect data. The research subjects included employees of knowledge-based enterprises in Shandong, Shanghai, Beijing, and Jiangsu Province. Further, the ethical approval for underlying research has obtained from Jiangsu Vocational College of Finance and Economics Research Ethics Review Committee. Moreover, the survey was carried out with due consent of participants.

Before the investigation began, the interviewees were informed of the purpose, process, and estimated completion time of this investigation. The investigation period was from June 2021 to August 2021. After excluding 18 invalid questionnaires, 392 valid questionnaires were obtained. Table 1 presents the demographic description of the sample.

**Table 1:** Demographic description of research sample (N = 392).

Variable	Category	Count	Proportion (%)
Gender	Male	187	47.7
	Female	205	52.3
Age	18–25	67	17.1
	25–35	225	57.4
	35–45	77	19.6
	45–55	20	5.1
	55 or above	3	0.8
Working years	Less than one year	59	15.1
	1–5 years	134	34.2
	5–10 years	109	27.8
	10–20 years	78	19.9
	20 or above	12	3.0
Level of education	High school	33	8.4
	college	64	16.3
	Undergraduate course	172	43.9
	master's degree	111	28.3
	doctor	12	3.1

### 3.2 Measuring tools

The measurement items were adapted based on prior research. The psychological contract violation was measured by the 20 item scale compiled by Ref. [44]. The overall job satisfaction scale of [45] was used for job satisfaction. The turnover intention scale by scholars [46]. The Global Measure scale of [47] was used for job embedding. We used a five-point Likert scale ranging from 1 (“strongly disagree”) to 5 (“strongly agree”).

### 3.3 Data analysis and results

#### 3.3.1 Reliability analysis

Table 2 shows the reliability test results for each variable. Table 1 shows that the Cronbach's alpha coefficient values corresponding to the four subscales are all greater than 0.8, and the CITC value of each measurement item exceeds 0.5, indicating that the internal consistency of the questionnaire is good and the results of this survey are highly reliable. However, the deleted Cronbach's alpha value of most items is lower than the Cronbach's alpha reliability coefficient in each dimension. All the questions are the measurement of the same concept, and there was no need to delete the items.

**Table 2:** Reliability test results.

Measurement items	CITC coefficient	α coefficient after deleting that item	Cronbach α coefficient
PCV1	0.629	0.924	0.928
PCV2	0.64	0.924	
PCV3	0.525	0.926	
PCV4	0.657	0.924	
PCV5	0.546	0.926	
PCV6	0.627	0.924	
PCV7	0.620	0.924	
PCV8	0.641	0.924	
PCV9	0.518	0.926	
PCV10	0.635	0.924	
PCV11	0.647	0.924	
PCV12	0.540	0.926	
PCV13	0.641	0.924	
PCV14	0.513	0.927	
PCV15	0.629	0.924	
PCV16	0.587	0.925	
PCV17	0.588	0.925	
PCV18	0.615	0.924	
PCV19	0.652	0.924	
PCV20	0.657	0.923	
JS1	0.608	0.824	0.844
JS2	0.687	0.803	
JS3	0.730	0.790	
JS4	0.590	0.828	
JS5	0.639	0.816	
JE1	0.689	0.870	0.888
JE2	0.666	0.873	
JE3	0.637	0.876	
JE4	0.691	0.870	
JE5	0.778	0.858	
JE6	0.660	0.874	
JE7	0.640	0.876	
TI1	0.653	0.867	0.882
TI2	0.685	0.862	
TI3	0.783	0.845	
TI4	0.659	0.866	
TI5	0.707	0.858	
TI6	0.659	0.866	

Note: Psychological contract violation(PCV); Turnover intention (TI); Job Satisfaction (JS); Job embeddedness (JE).

### 3.4 Validity analysis

The 20 items of psychological contract violation are extracted into six factors, as follows: development opportunities, good employment relationships, work itself, income, benefits, and provision of resources. The dimensions of each construct are consistent with the theoretical dimensions of the [44] scale. Job satisfaction, turnover intention, and job embeddedness are analyzed as single factors.

As Table 3 shows, in this research model, all values of CMIN/DF, NFI, IFI, TLI, CFI, GFI and RMSEA are highly consistent with the suggested value. Where a better fitness index of this model also indicates a relatively stronger convincingness of the relationship among the variables.

**Table 3:** Measurement model fitting index value.

CMIN	DF	CMIN/DF	NFI	IFI	TLI	CFI	GFI	RMSEA
981.503	629	1.560	0.886	0.956	0.950	0.955	0.888	0.038
Suggested value		<3	>0.8	>0.9	>0.8	>0.9	>0.8	<0.08

Note: GFI (Goodness-of-Fit Index), RMSEA (Root Mean Square Error of Approximation), TLI (Tucker-Lewis Index), CFI (Comparative Fit Index), NFI (Normal Fitting Index), IFI (Incremental Fitting Index).

This study uses the Composite Reliability (CR) value and the average variance extraction (AVE) value as the evaluation criteria of convergent validity. When the CR value of each factor is greater than 0.7 and the AVE value is greater than 0.50, it is generally considered that the convergent validity is better. Table 4 shows that the factor loading of each measured variable is greater than 0.6, and is significant at the 0.001 level, indicating that the measured variable has strong explanatory power for each latent variable. The combined reliability of each latent variable is greater than 0.6, indicating that the correlation between the measured variables of each latent variable is strong, and the internal consistency is good. The AVE value is greater than 0.5, indicating that the latent variable has a strong explanatory power for its corresponding measurement variable. The measured variable of each latent variable can converge at one point. Therefore, the degree of convergence is good.

**Table 4:** Convergent validity.

Variable relationship			Coefficient estimates	Standard error	Critical value	P	Factor loading	AVE	CR
PCV1	<---	Welfare	1.000				0.801	0.640	0.842
PCV2	<---	Welfare	0.968	0.059	16.376	***	0.818		
PCV3	<---	Welfare	1.049	0.067	15.685	***	0.781		
PCV4	<---	Income	1.000				0.787	0.622	0.832
PCV5	<---	Income	1.158	0.075	15.487	***	0.799		
PCV6	<---	Income	1.013	0.067	15.157	***	0.780		
PCV7	<---	Development opportunities	1.000				0.839	0.683	0.866
PCV8	<---	Development opportunities	1.015	0.057	17.751	***	0.816		
PCV9	<---	Development opportunities	1.098	0.061	17.932	***	0.824		
PCV10	<---	Job itself	1.000				0.804	0.645	0.879
PCV11	<---	Job itself	0.999	0.057	17.390	***	0.815		
PCV12	<---	Job itself	1.105	0.064	17.204	***	0.808		
PCV13	<---	Job itself	0.971	0.059	16.576	***	0.784		
PCV14	<---	Work resources provided	1.000				0.806	0.645	0.845
PCV15	<---	Work resources provided	0.873	0.053	16.333	***	0.820		
PCV16	<---	Work resources provided	0.841	0.054	15.683	***	0.783		
PCV17	<---	Good employment relationship	1.000				0.838	0.633	0.873
PCV18	<---	Good employment relationship	0.835	0.049	17.086	***	0.776		
PCV19	<---	Good employment relationship	0.851	0.049	17.509	***	0.790		
PCV20	<---	Good employment relationship	0.820	0.048	17.134	***	0.777		
JS1	<---	Job satisfaction	1.000				0.684	0.525	0.846
JS2	<---	Job satisfaction	1.098	0.085	12.989	***	0.762		
JS3	<---	Job satisfaction	1.188	0.088	13.460	***	0.798		
JS4	<---	Job satisfaction	0.918	0.080	11.510	***	0.661		
JS5	<---	Job satisfaction	0.986	0.080	12.255	***	0.710		
JE1	<---	Job embeddedness	1.000				0.731	0.532	0.888
JE2	<---	Job embeddedness	0.980	0.072	13.557	***	0.712		
JE3	<---	Job embeddedness	0.905	0.069	13.035	***	0.686		
JE4	<---	Job embeddedness	1.031	0.073	14.102	***	0.740		
JE5	<---	Job embeddedness	1.285	0.081	15.905	***	0.836		
JE6	<---	Job embeddedness	0.902	0.068	13.310	***	0.700		
JE7	<---	Job embeddedness	0.882	0.067	13.139	***	0.691		
TI1	<---	Turnover intention	1.000				0.703	0.558	0.883
TI2	<---	Turnover intention	1.098	0.081	13.483	***	0.740		
TI3	<---	Turnover intention	1.351	0.090	15.006	***	0.833		
TI4	<---	Turnover intention	1.042	0.080	13.058	***	0.715		
TI5	<---	Turnover intention	1.173	0.084	14.009	***	0.771		
TI6	<---	Turnover intention	1.025	0.079	12.981	***	0.710		

Discriminant validity indicates the correlation in the internal data of the latent variable. If the square root of AVE is greater than the absolute value of the correlation coefficient between the latent variables, it means that the internal correlation is greater than the external correlation, indicating that there is a difference between the latent variables and that the discriminant validity is high. Table 5, the table of discriminant validity shows that the AVE value of each latent variable is greater than the square of the correlation coefficient between the latent variables; that is, it can meet the standard that the square root of AVE is greater than the correlation coefficient, indicating that the internal correlation of the latent variables is greater than the external correlation, and the discriminant validity between latent variables is high.

**Table 5:** Discriminant validity.

	Welfare	Income	Development opportunities	Job itself	Work resources provided	Good employment relationship	Job satisfaction	Job embeddedness	Turnover intention
Welfare	0.800								
Income	0.603	0.789							
Development opportunities	0.482	0.559	0.826						
Job itself	0.596	0.600	0.505	0.803					
Work resources provided	0.564	0.558	0.498	0.520	0.803				
Good employment relationship	0.571	0.597	0.612	0.542	0.542	0.796			
Job satisfaction	−0.385	−0.270	−0.284	−0.245	−0.279	−0.249	0.725		
Job embeddedness	−0.186	−0.177	−0.151	−0.157	−0.220	−0.166	0.295	0.730	
Turnover intention	0.328	0.294	0.360	0.376	0.303	0.342	−0.466	−0.078	0.747

### 3.5 Descriptive statistics and correlation analysis

Table 6 presents the mean value, standard deviation, correlation coefficient, and internal consistency coefficient of each variable. The results show that psychological contract violation and turnover intention (r = 0.406, p < 0.01) are positively correlated; psychological contract violation and job satisfaction (r = −0.335, p < 0.01) are negatively correlated; job satisfaction and job embeddedness (r = −0.263, p < 0.01) are negatively correlated; job satisfaction and turnover intention (r = −0.427, p < 0.01) are negatively correlated. This provides a good foundation for the following hypothesis verification.

**Table 6:** Pearson related.

	Average	Standard deviation	Job satisfaction	Job embeddedness	Turnover intention	Psychological contract violation
Job satisfaction	3.495	0.869	1			
Job embeddedness	3.451	0.874	0.263**	1		
Turnover intention	3.290	0.917	−0.427**	−0.073	1	
Psychological contract violation	3.293	0.807	−0.335**	−0.201**	0.406**	1

*p < 0.05 ** p < 0.01.

### 3.6 Hypothesis test

Table 7 shows the results of the main effect analysis. Psychological contract violation has a significant effect on turnover intention (P < 0.01), and the standardized path coefficient value is 0.313, which means that psychological contract violation has a positive effect on turnover intention. Path 1 is supported. Therefore, Hypothesis 1 is verified, and psychological contract violation has a positive effect on turnover intention. Psychological contract violation has a significant effect on job satisfaction (P < 0.01), and the standardized path coefficient value is −0.383, which means that psychological contract violation has a negative effect on job satisfaction. Path 2 is supported. Therefore, Hypothesis 2 is verified, and employees’ psychological contract violations have a negative effect on job satisfaction. Job satisfaction has a significant effect on turnover intention (P < 0.01), and the standardized path coefficient value is −0.347, which means that job satisfaction has a negative effect on turnover intention. Path 3 is supported. Therefore, Hypothesis 3 is verified, and job satisfaction has a negative effect on turnover intention.

**Table 7:** Path analysis.

Path	Relationship path between variables			Standardized regression coefficient	T value	P	Hypothesis test results
H1	Psychological contract violation	─→	Turnover intention	0.313	5.011	***	supported
H2	Psychological contract violation	─→	Job satisfaction	−0.383	−5.863	***	supported
H3	Job satisfaction	─→	Turnover intention	−0.347	−5.576	***	supported

Table 8 shows that through the analysis of each mediating path of the model, the indirect effect bias correction confidence interval and test significance corresponding to each path are shown in the table above. In the path of “turnover intention-job satisfaction-turnover intention,” the deviation correction value CI based on the mediating effect of job satisfaction is [0.079, 0.204], the interval does not contain 0, and the P value is less than 0.05; that is, the indirect effect of the path is significant and there is a mediating effect.

**Table 8:** Mediating effect test.

Path	Mediating variable	Indirect effect		
		Boot CI upper limits	Boot CI lower limits	Significance
Psychological contract violation─→	Job satisfaction	0.079	0.204	0.000
Job satisfaction─→Turnover intention				

Table 9 shows that through the decomposition of the total effect, direct effect, and mediating effect, in the path of “psychological contract violation-job satisfaction-turnover intention,” the relative effect value of the direct effect is 70.18% and the relative effect value of the mediating effect is 29.82%. This indicates that the mediating effect of this path is a partial mediating effect, whereby psychological contract violation affects turnover intention through the mediating effect of job satisfaction. Therefore, Hypothesis 4 is verified, and job satisfaction plays a mediating role between psychological contract violation and turnover intention.

**Table 9:** Decomposition of total effect, direct effect, and mediating effect.

Path	Effect	Effect value	Corresponding effect value
Psychological contract violation─→	Total effect	0.446	
Job satisfaction─→Turnover intention	Direct effect	0.313	70.18%
	Mediating effect	0.133	29.82%

Table 10 shows that the results of applying SPSS Model 5, compiled by Hayes (2012), to conduct a moderated mediating effect test, show that in the model with psychological contract violation, job satisfaction, job embeddedness, and the interaction between psychological contract violation and job embeddedness as independent variables and turnover intention as dependent variables, the R^2^ is 0.289, the F-value is 15.449, and the job embeddedness coefficient is positive, passing the significance test, indicating a positive effect. The interaction coefficient between psychological contract violation and job embeddedness is negative, passing the significance test, and the non-standardized regression coefficient value of the interaction term is opposite to the coefficient value of the interaction term, indicating that there is a negative moderating effect. Therefore, Hypothesis 5 is verified. Job embeddedness plays a moderating role in the influential process of psychological contract violation on the turnover intention of knowledge workers.

**Table 10:** Model test.

	Job satisfaction	Turnover intention
	B	B
Constant	3.4892	4.547
Gender	YES	YES
Age	YES	YES
Level of education	YES	YES
Working years	YES	YES
Psychological contract violation	−0.363	0.416
Job satisfaction		−0.359
Job embeddedness		0.122
Psychological contract violation *Job embeddedness		−0.187
Sample amount	392	392
R^2^	0.124	0.289
F value	F = 7.771, p = 0.000	F = 15.449, p = 0.000

*p < 0.05 ** p < 0.01.

For the path empirical results, refer to Table 11, which present a summary of the hypotheses verification tests.

**Table 11:** Summary of hypotheses testing.

Hypothesis	Content	Verification
H1	Knowledge workers' psychological contract violation has a positive effect on turnover intention.	Supported
H2	Employees' psychological contract violations have a negative impact on job satisfaction.	Supported
H3	Job satisfaction has a negative impact on turnover intention.	Supported
H4	Job satisfaction plays a mediating role between psychological contract violation and turnover intention.	Supported
H5	Job embeddedness plays a moderating role in the influential process of psychological contract violation on knowledge workers' turnover intention.	Supported

## 4 Discussion and conclusion

First, psychological contract violation of knowledge workers has a significant direct effect on turnover intention and job satisfaction. This study researched the direct effects of psychological contract violation on turnover intention, psychological contract violation on job satisfaction, and job satisfaction on turnover intention. It found that psychological contract violation has significant positive effects on turnover intention, and psychological contract violation has significant negative effects on job satisfaction. Therefore, reducing employees' psychological contract violations has a significant effect on improving employees’ job satisfaction and reducing turnover intention as it was investigated earlier by Ref. [8].

Second, job satisfaction plays a mediating role between psychological contract violation and turnover intention. Job satisfaction plays a partial mediating role between psychological contract violation and turnover intention behavior. In other words, psychological contract violation can affect turnover intention directly, and it can also have an indirect effect on employees’ job withdrawal behavior through job satisfaction. Likewise, job satisfaction has already been investigated in Chinese educational sector in order to give indications for other sectors to explore it in China [22].

Finally, job embeddedness plays a moderating role in the influential process of psychological contract violation on the turnover intention of knowledge workers. Job embeddedness negatively regulates the influence of psychological contract violation on turnover intention. In other words, the higher the degree of job embeddedness, the weaker the positive impact of psychological contract violation on turnover intention. Job embeddedness also negatively regulates the mediating role of job satisfaction, whereby the higher the degree of job embeddedness, the weaker the mediating role of job satisfaction. Similarly, [13]; has explained job embeddedness in China hence addressed certain guidelines for human resource to be cautious about turnover intentions of employees.

## 5 Management implications

The management implications of this study lie in the following aspects:

Management should identify the special group of knowledge workers and formulate a management model that benefits the enterprise and the employees. Reasonable identification of knowledge workers can help an enterprise to lock in special management groups. Understanding the characteristics of this type of talent will help organizations formulate a management system that is more suitable for this type of employee. Effective incentive measures will help skilled knowledge workers achieve their personal potential and maximize their self-realization, resulting in a win-win situation for both parties. A suitable management model will help an enterprise to achieve its business objectives, accumulate reproducible and continuously improved management experience, reduce the risk of gaps in the system and lack of talent structure, and help enterprises to improve their market competitive advantages and achieve rapid and stable development.

The source of the psychological contract should be controlled. To achieve a unified approach in all aspects, including employee entry, training, organizational culture, and management systems, enterprises should pay special attention to the standardization of their employee manual. Employees have a variety of perspectives on organizational agents. These agents can include human resources department recruiters, human resources managers, senior organizational leaders, direct department leaders, leaders of other departments, and senior employees. The organizational responsibilities mentioned in different sources of information become the responsibilities that the organization should perform as perceived by the employees. When an organization fails to fulfill these responsibilities, the result is employees’ psychological contract violation.

Job embeddedness provides a new perspective for human resource management. Job embeddedness is distinguished from subjective factors such as job satisfaction and organizational commitment. It begins with objective factors such as organizational level and community, focusing on the degree of embeddedness between individuals and jobs. In the context of a transitional economy, job embeddedness enables managers to explain and predict employee behaviors more comprehensively. Then, they can take corresponding management measures to reduce employee turnover and the cost of human resources management, including recruitment and training. Therefore, the core competitiveness of enterprises can be further improved. In an increasingly competitive talent market, employees who encounter psychological contract violations may not necessarily choose to withdraw from or ignore behaviors. The motivation for individuals to stay in the organization is often related to family, work teams, and colleagues. The stronger the connection, the higher the degree of job embeddedness, and the greater the sense of belonging the employee has to the enterprise and the community.

## 6 Deficiencies and prospects

Future studies on the outcome variables of psychological contract violations could focus on specific professional fields. Differences in employment conditions and the content of the psychological contract mean that the types and dimensions of the psychological contract will also be different. The measurement of psychological contract violation should be closer to the reality of the organization, whereby management theory is closer to the practice of the organization, making it instructive and operable. While the mediating effect and moderating effect of this study have been verified, there may be other influence paths. Therefore, future studies should explore more paths of psychological contract violations that affect turnover intention and ways to reduce the impact of psychological contract violations.

## Author contribution statement

Zhenhua He: Conceived and designed the experiments; Performed the experiments; Analyzed and interpreted the data; Wrote the paper.

Lifeng Chen: Performed the experiments; Analyzed and interpreted the data; Contributed reagents, materials, analysis tools or data; Wrote the paper.

Zahid Shafait: Conceived and designed the experiments; Analyzed and interpreted the data.

## Funding statement

This work was supported by 10.13039/100014473Shandong Education Science Plan [2021ZC109].

## Data availability statement

Data will be made available on request.

## Declaration of interest's statement

The authors declare the following conflict of interests: Lifeng Chen has two affiliations: School of Public Affairs, Zhejiang University, Hangzhou 310058, Zhejiang, China and School of Business, Zhejiang University City College, Hangzhou 310015, Zhejiang, China. Further, Zahid Shafait is switching his educational institute and author will provide the information regarding his next educational institute and email address once it is done.
